# Divergent pathways from body image to disordered eating and physical activity: differential roles of thin-ideal and muscular-ideal internalization among Chinese female college students

**DOI:** 10.3389/fpsyg.2026.1823005

**Published:** 2026-09-16

**Authors:** Hongquan Chen, Qian Li, Hanru Li, Han Liu, Hongyao Li, Yitong Wang, Jiaqi Xue, Meng Wang

**Affiliations:** 1 College of Physical Education and Health, Yancheng Teachers University, Yancheng, China; 2 College of Sports Science, Shenyang Normal University, Shenyang, China; 3 Martial Arts and Dance Academy, Shenyang Sport University, Shenyang, China; 4 Department of Rehabilitation, Longgang Central Hospital, Shenzhen, China; 5 First Clinical College, China Medical University, Shenyang, China; 6 Graduate School Division of Sports Science, Sendai University, Sendai, Japan

**Keywords:** body image, dual-pathway model, eating disorders, muscular-ideal internalization, physical activity, thin-ideal internalization

## Abstract

**Objective:**

To construct and test a dual-pathway differentiation model exploring associations of body image with eating disorders and physical activity among female college students, examining the differential mediating roles of thin-ideal internalization and muscular-ideal internalization.

**Methods:**

Using a cross-sectional design, data were collected from 4,172 female college students across six universities in mainland China via online questionnaires. Structural equation modeling was employed to examine patterns of association among body image, thin-ideal internalization, muscular-ideal internalization, depression, perceived body fat, eating disorders, and physical activity. The bias-corrected Bootstrap method (5,000 resamples) was used to test mediating effects.

**Results:**

The structural equation model showed a good fit (RMSEA = 0.044). All mediating and direct pathways in the model reached statistical significance. Thin-ideal internalization played a chain-mediating role between body image and eating disorders, while muscular-ideal internalization independently mediated the association between body image and physical activity.

**Conclusions:**

Body image can differentiate into risk-oriented and adaptation-oriented behavioral pathways through different types of ideal internalization. Body image was associated with eating disorder risk, whereas muscular-ideal internalization was associated with engagement in physical activity. These findings indicate that the type of body ideal internalization functions as a core mechanism of behavioral differentiation, providing a theoretical basis and potential intervention targets for eating disorder prevention and health behavior promotion.

## 1 Introduction

Body image is a multidimensional and comprehensive concept encompassing an individual's perceptions of their own appearance as well as emotional and behavioral responses (Cash, 2004). Body image can manifest in positive forms and negative characteristics (Tylka and Wood-Barcalow, 2015). Body Image Disturbance (BID) refers to perceptual biases, behavioral abnormalities, and cognitive distortions related to weight and body shape, with its core feature being persistent negative attitudes and evaluations toward one's own body (Ainley and Tsakiris, 2013). Among female populations, BID has been established as a critical risk factor in the development of eating disorders (Ricciardelli and McCabe, 2004). Female college students are a particularly vulnerable population for body image concerns, as emerging adulthood is marked by heightened social comparison, identity exploration, and susceptibility to sociocultural appearance pressures (Barbierik et al., 2023), with peak onset of disordered eating occurring during this developmental stage (Eisenberg et al., 2011). In China, nearly 70% of college students have engaged in weight loss behaviors (Wang et al., 2023), and female students consistently report higher levels of body dissatisfaction, depression, and emotional eating compared to their male counterparts (Wu et al., 2023). This evidence underscores the need for empirical investigation into the mechanisms by which sociocultural pressures shape behavioral outcomes in this understudied population.

Research evidence indicates that compared with men, women report higher levels of BID and related eating disorder symptoms (Gu et al., 2025; Guo et al., 2022). This gender difference may be partially explained by women's tendency to select slimmer body shapes as ideal body standards (Deshmukh and Kulkarni, 2017), and is also reinforced by social pressures from parents, peers, and media (Tort-Nasarre et al., 2021). These factors work in synergy, increasing vulnerability to the internalization of negative emotions such as depression and shame, thereby exacerbating BID (Hu et al., 2024).

Building on this foundation, BID is associated with eating disorder behaviors, and these associations appear to operate through thin-ideal internalization and cognitive-emotional processes such as negative emotions, attentional bias, and selective memory (Tamburrino, 2003). The dual-pathway framework tested in this study builds upon Tylka's (2011) model—originally developed for men—by extending it to female populations, incorporating depressive symptoms and weight perception as mediators, and simultaneously testing both thin-ideal and muscular-ideal internalization within a unified model (Tylka, 2011). However, the relationship between body image and behavioral outcomes is not limited to a single negative pathway. Attention to one's body can also be transformed into motivation to improve physical condition, motivating individuals to engage in physical activity (Ouyang et al., 2020). Empirical research has also found that physical activity participation is associated with higher levels of body appreciation (Abu Alwafa and Badrasawi, 2023), while being related to improved body image satisfaction among women (Leng et al., 2020). These findings thus suggest the possibility of a virtuous cycle in which physical activity participation may contribute to BID reduction (Gao et al., 2024). However, it is important to acknowledge that this pathway is not universally adaptive. Excessive pursuit of muscularity can lead to compulsive exercise, muscle dysmorphia, and supplement misuse.

Therefore, research on female college students raises a core question: when individuals already experience BID, why does it differentiate into two distinct behavioral pathways—risk-oriented (eating disorders) and adaptation-oriented (physical activity participation)? This study proposes that the degree to which individuals internalize different body ideals is the key differentiation mechanism guiding the transformation of body image experiences into different behavioral outcomes.

Specifically, when individuals highly internalize the thin-ideal, a risk-oriented pathway centered on weight control may emerge: by reinforcing an extreme pursuit of a slender body type, thin-ideal internalization may heighten weight perception and induce depressive emotions (El Ansari and Berg-Beckhoff, 2019; Tang et al., 2009). Thin-ideal and muscular-ideal internalization work in synergy and jointly contribute to eating disorder behaviors, including restrictive eating and binge eating (Stice et al., 2011). Conversely, when individuals highly internalize the muscular-ideal, an adaptation-oriented pathway focused on functional enhancement may emerge: muscular-ideal internalization can be transformed into positive pursuit of a fit body type, motivating individuals to engage more actively in physical activity (Homan, 2010; Soulliard et al., 2019).

A comprehensive analysis of existing research reveals three main limitations. First, most studies examine the relationship between a single body ideal internalization (typically thin-ideal internalization) and a single outcome variable in isolation (Fitzsimmons-Craft et al., 2012), failing to simultaneously test the differential effects and interactions of both types of ideal internalization within the same model. Second, explorations of mediating mechanisms are often limited to single mediation pathways (e.g., examining only depression or BID), and it remains unclear how depressive emotions and body-related cognitive evaluations (such as perceived body fat) synergistically shape behavioral outcomes through multiple pathways (Rodgers et al., 2015). Finally, the adaptive pathway—specifically, whether muscular-ideal internalization can initiate a health-promoting pathway toward sustained physical activity, rather than contributing to risk—lacks systematic validation and remains underexplored (Araiza and Freitas, 2024).

Therefore, this study constructs and validates a dual-pathway differentiation model aimed at addressing the aforementioned theoretical gaps. The model has the following core features: using body image as a latent variable and core starting point; employing thin-ideal internalization and muscular-ideal internalization as key differentiation mechanisms to reveal how body image experiences diverge into different pathways; in the risk pathway, examining whether body image is associated with eating disorders via thin-ideal internalization, depressive emotions, and perceived body fat; in the adaptive pathway, examining whether body image is associated with physical activity participation through muscular-ideal internalization.

Based on the theoretical framework above, this study proposes the following hypotheses: Hypothesis 1 (Risk Pathway): Body image is indirectly associated with eating disorders through associations with thin-ideal internalization (H1a), depression (H1b), and perceived body fat (H1c). Body image is also directly associated with eating disorders (H1d). Hypothesis 2 (Adaptive Pathway): Body image is indirectly associated with physical activity participation through its association with muscular-ideal internalization (H2a). Body image is also directly associated with physical activity participation (H2b). Hypothesis 3 (Exploratory Hypothesis): There is a significant correlation between thin-ideal internalization and muscular-ideal internalization (H3).

## 2 Methods

### 2.1 Study design and ethical approval

This study was approved by the ethical committee of the Institute of Psychology, Chinese Academy of Sciences (approval number: H25145) and was conducted in accordance with national and institutional requirements. All participants were informed of the research purpose, data usage, and privacy protection measures before completing the questionnaire and provided written informed consent. As all participants were 18 years of age or older, parental or legal guardian consent was not required.

### 2.2 Participants and data collection

Using convenience sampling, data were collected from October 4–December 4, 2024, via an online survey platform (Wenjuanxing). Participants were female college students from six universities in mainland China, covering different regions, including North China, East China, and Northeast China, providing a degree of representativeness. The demographic section collected sociodemographic information, including age (in years), height (in meters), and weight (in kilograms) from participants. Height and weight were collected primarily for descriptive purposes to characterize the sample, rather than as substantive variables in the model. To ensure data quality, invalid questionnaires were excluded based on the following criteria: response time less than 3.5 min or exceeding 30 min; obvious patterned responses (e.g., selecting the same option throughout); logical contradictions (e.g., inconsistencies in key items). Participants received no monetary compensation for their participation.

### 2.3 Measurement instruments

All scales used in this study were administered in their validated Chinese-translated versions, with satisfactory reliability and validity previously established in Chinese populations.

### 2.4 Sociocultural pressure

Sociocultural pressure perception was measured using the pressure dimension of the Sociocultural Attitudes Toward Appearance Questionnaire-4 (SATAQ-4) (Neves et al., 2020), which comprises three dimensions: family pressure, peer pressure and media pressure, reflecting appearance evaluation pressure and ideal body type requirements perceived by individuals from family members, peers and media, respectively. The scale uses a 5-point Likert scale (1 = strongly disagree, 5 = strongly agree), with higher scores indicating greater perceived sociocultural pressure. In this study, Cronbach's α coefficients for each dimension were 0.838 (family), 0.927 (peer), and 0.967 (media), indicating good internal consistency.

### 2.5 Thin-ideal internalization

Thin-ideal internalization was measured using the thin-ideal internalization subscale of the Sociocultural Attitudes Toward Appearance Questionnaire-4 (SATAQ-4) (Neves et al., 2020), reflecting the degree to which individuals accept and pursue slender body standards. The scale uses a 5-point Likert scale (1 = strongly disagree, 5 = strongly agree), with higher scores indicating higher levels of thin-ideal internalization. In this study, Cronbach's α for this subscale was 0.743, indicating acceptable reliability.

### 2.6 Muscular-ideal internalization

Muscular-ideal internalization was measured using the muscular-ideal internalization subscale of the Sociocultural Attitudes Toward Appearance Questionnaire-4 (SATAQ-4) (Neves et al., 2020), reflecting the degree to which individuals accept and pursue fit and strong body standards. The scale uses a 5-point Likert scale (1 = strongly disagree, 5 = strongly agree), with higher scores indicating higher levels of muscular-ideal internalization. In this study, Cronbach's α for this subscale was 0.842, indicating good reliability.

### 2.7 Perceived body fat

Perceived body fat was measured using the obesity perception dimension of the Chinese version of the Physical Self-Description Questionnaire (PSDQ) (Yang, 2002), reflecting the degree of negative evaluation of one's own weight and body shape. The scale uses a 6-point scale (1 = does not apply at all, 6 = applies completely), with higher scores indicating stronger obesity perception. In this study, Cronbach's α for this dimension was 0.919, indicating excellent reliability.

### 2.8 Physical activity

Physical activity participation was measured using the physical activity dimension of the Chinese version of the Physical Self-Description Questionnaire (PSDQ) (Yang, 2002), reflecting the frequency and intensity of individuals' daily participation in physical exercise. The scale uses a 6-point scale (1 = does not apply at all, 6 = applies completely), with higher scores indicating higher levels of physical activity participation. In this study, Cronbach's α for this dimension was 0.718, indicating acceptable reliability.

### 2.9 Depressive symptoms

Depressive emotions were measured using the Patient Health Questionnaire-9 (PHQ-9) (Kroenke et al., 2001), which contains nine items assessing the frequency of depressive symptoms over the past 2 weeks. The scale uses a 4-point scale (1 = not at all, 4 = nearly every day), with higher total scores indicating more severe depressive symptoms (Yu et al., 2025). In this study, Cronbach's α for this scale was 0.919, indicating excellent reliability and good structural validity.

### 2.10 Disordered eating

Disordered eating was measured using the Eating Disorder Examination Questionnaire-Short (EDE-QS) (He et al., 2021), which contains 12 items assessing the frequency of restrictive eating, binge eating, and appearance-weight concerns over the past 7 days. The scale uses a 4-point scale (1 = 0 days, 4 = 6–7 days), with higher total scores indicating greater severity of eating disorders (Tian et al., 2025). In this study, Cronbach's α for this scale was 0.918, indicating excellent reliability.

## 3 Data analysis

All statistical analyses were conducted using SPSS 26.0 (IBM, Armonk, NY, USA) and AMOS 26.0 (IBM, Armonk, NY, USA). The significance level for all statistical tests was set at α = 0.05.

### 3.1 Common method bias test

Given that all variables were measured using self-report questionnaires, Harman's single-factor test was applied to assess potential common method bias (Podsakoff et al., 2003). An unrotated exploratory factor analysis (EFA) was conducted on all measurement items. If the first factor explained less than 40% of the total variance, below the critical threshold, this indicates that common method bias does not constitute a significant problem in this study.

### 3.2 Descriptive statistics and correlation analysis

Pearson correlation analysis examined bivariate relationships among major study variables, including family pressure, peer pressure, media pressure, thin-ideal internalization, muscular-ideal internalization, depression, perceived body fat, disordered eating, and physical activity. Following Cohen (1988) conventions, correlation coefficients of 0.10, 0.30, and 0.50 were interpreted as small, medium, and large effect sizes, respectively.

### 3.3 Structural equation modeling

AMOS 26.0 was used to construct a structural equation model (SEM) to test the hypothesized dual-pathway differentiation model. The model was constructed as follows. First, body image was modeled as a latent variable with family pressure, peer pressure, and media pressure as observed indicators. The latent variable was operationalized using family, peer, and media pressure subscales from the SATAQ-4, consistent with prior SEM studies that have employed these sociocultural pressures as indicators of appearance-related concerns (Frederick et al., 2022; Roberts et al., 2022). This approach reflects the tripartite influence model's conceptualization of these pressures as the sociocultural context within which body image is shaped, rather than as direct measures of body image satisfaction. The model included two core pathways: the risk pathway, examining associations between body image and disordered eating, with thin-ideal internalization, depression, and perceived body fat as potential linking variables; the adaptive pathway, examining the association between body image and physical activity participation, with muscular-ideal internalization as a potential linking variable. The model allowed a covariance relationship between thin-ideal internalization and muscular-ideal internalization. Model estimation employed the maximum likelihood method. Model fit was comprehensively evaluated using multiple indices: Comparative Fit Index (CFI > 0.90 acceptable, > 0.95 good), Tucker-Lewis Index (TLI > 0.90 acceptable, > 0.95 good), Root Mean Square Error of Approximation (RMSEA < 0.08 acceptable, < 0.06 good), and Standardized Root Mean Square Residual (SRMR < 0.08 acceptable, < 0.05 good) (Hu and Bentler, 1999). Given that the chi-square statistic (χ^2^) and its ratio to degrees of freedom (χ^2^/df) are highly sensitive to sample size and may yield significant χ^2^ values even when model fit is good in large-sample studies (Wang, 2014), this study placed greater emphasis on the comprehensive performance of incremental and absolute fit indices.

### 3.4 Mediation and direct effect testing

The bias-corrected percentile Bootstrap method was used to test the significance of effects, with 5,000 resamples and 95% confidence intervals (CIs) (Preacher and Hayes, 2008). Hypothesis 1 tested the mediating effects of body image on disordered eating through thin-ideal internalization (H1a), depression (H1b), and perceived body fat (H1c), as well as the direct effect of body image on disordered eating (H1d). For Hypothesis 2 (adaptive pathway), the mediating effect of body image on physical activity participation through muscular-ideal internalization (H2a), as well as the direct effect of body image on physical activity (H2b), were tested. When the 95% CI did not include 0, the effect was considered statistically significant. Direct effects, indirect effects, and total effects were reported, along with their standardized coefficients (β), standard errors (SE), and confidence intervals (CI).

## 4 Results

### 4.1 Sample screening and descriptive statistics

A total of 8,951 questionnaires were collected in this study, with 4,172 valid participants ultimately included, yielding an effective response rate of 46.6%. The mean age of participants was 20.3 ± 1.5 years, with an age range of 18–25 years. The mean body mass index (BMI) was 21.82 ± 5.41 kg/m^2^.

### 4.2 Common method bias test

Harman's single-factor test was adopted to evaluate common method bias derived from self-reported questionnaires. The unrotated exploratory factor analysis revealed that the variance explained by the first extracted common factor accounted for only 28.37% of the total variance, which fell below the critical cutoff value of 40%. Accordingly, severe common method bias was not detected in the present dataset.

### 4.3 Descriptive statistics and correlation analysis

Table 1 presents the means, standard deviations, and correlation coefficients of all study variables. Results indicated that disordered eating was significantly positively correlated with all predictor variables, including thin-ideal internalization (*r* = 0.455, *p* < 0.01), muscular-ideal internalization (*r* = 0.342, *p* < 0.01), family pressure (*r* = 0.458, *p* < 0.01), peer pressure (*r* = 0.506, *p* < 0.01), media pressure (*r* = 0.505, *p* < 0.01), perceived body fat (*r* = 0.624, *p* < 0.01), and depression (r = 0.431, *p* < 0.01), with a weaker positive correlation with physical activity (*r* = 0.191, *p* < 0.01).

**Table 1:** Descriptive statistics and correlation analyses of main variables.

Variables	M ±SD	1	2	3	4	5	6	7	8	9
1 Eating disorders	1.507 ± 0.572	1								
2 Thin-ideal internalization	2.954 ± 0.760	0.455^**^	1							
3 Muscular-ideal internalization	2.400 ± 0.812	0.342^**^	0.719^**^	1						
4 Family	2.476 ± 0.967	0.458^**^	0.401^**^	0.369^**^	1					
5 Peers	2.389 ± 1.033	0.506^**^	0.463^**^	0.410^**^	0.668^**^	1				
6 Media	2.828 ± 1.192	0.505^**^	0.476^**^	0.369^**^	0.501^**^	0.614^**^	1			
7 Perceived body fat	2.949 ± 1.225	0.624^**^	0.409^**^	0.262^**^	0.569^**^	0.590^**^	0.512^**^	1		
8 Physical activity	2.922 ± 0.898	0.191^**^	0.251^**^	0.343^**^	0.263^**^	0.256^**^	0.171^**^	0.240^**^	1	
9 Depression	1.287 ± 0.470	0.431^**^	0.143^**^	0.073^**^	0.181^**^	0.220^**^	0.261^**^	0.277^**^	0.024	1

^**^p < 0.01.

Thin-ideal internalization and muscular-ideal internalization showed a strong positive correlation (*r* = 0.719, *p* < 0.01), supporting Hypothesis H3. Thin-ideal internalization and muscular-ideal internalization constructs were significantly positively correlated with family, peer, and media pressures, perceived body fat, and physical activity, but showed weaker correlations with depression (r = 0.143 and 0.073 respectively, *p* < 0.01). Family, peer, and media pressures showed moderate to strong positive intercorrelations (*r* = 0.501–0.668, *p* < 0.01), indicating that these pressure sources tend to covary in individuals' experiences. Perceived body fat was significantly positively correlated with all three forms of social pressures, depression, and physical activity. The correlation between physical activity and depression was not significant (*r* = 0.024, *p* > 0.05).

### 4.4 Construction and fit of the SEM

This study examined patterns of association among body image, disordered eating, and physical activity using a structural equation model by constructing an SEM. First, a latent body image variable was constructed with family pressure, peer pressure, and media pressure as its observed indicators. To optimize model fit and after consulting modification indices while ensuring theoretical reasonableness, covariance relationships were added between selected error terms in the model. The final model fit indices were as follows: χ^2^ = 136.108 (df = 15, *p* < 0.001), χ^2^/df = 9.074, CFI = 0.992, TLI = 0.981, RMSEA = 0.044 (90% CI: 0.037–0.051). Although χ^2^/df = 9.074 exceeded the traditional standard, considering the sensitivity of the chi-square statistic to large-sample sizes (*N* = 4,172) (Wang, 2014), and given that all other fit indices reached good standards, the model demonstrated good fit with the data and was acceptable.

## 5 Path analysis: direct and indirect effects

### 5.1 Predictive effects of body image

Body image significantly predicted thin-ideal internalization (β = 0.559, *p* < 0.001), muscular-ideal internalization (β = 0.514, *p* < 0.001), depression (β = 0.296, *p* < 0.001), and the three dimensions of sociocultural pressure perception, family pressure (β = 0.731, *p* < 0.001), peer pressure (β = 0.779, *p* < 0.001), and media pressure (β = 0.693, *p* < 0.001).

### 5.2 Prediction of outcome variables by mediating variables

Risk pathway: Thin-ideal internalization (β = 0.145, *p* < 0.001), depression (β = 0.248, *p* < 0.001), and perceived body fat (β = 0.274, *p* < 0.001) all significantly predicted disordered eating. Adaptive pathway: Muscular-ideal internalization predicted physical activity (β = 0.233, *p* < 0.001).

### 5.3 Direct effects

Body image demonstrated a significant direct effect on disordered eating (β = 0.293, *p* < 0.001), supporting Hypothesis H1d. Body image demonstrated a significant direct effect on physical activity (β = 0.216, *p* < 0.001), supporting Hypothesis H2b.

### 5.4 Testing of mediating effects

The bias-corrected Bootstrap method (5,000 resamples) was used to test specific mediating effects, with results presented in Table 2 and the model relationship diagram shown in Figure 1.

**Table 2:** Bootstrap test results for mediation effects.

Path	Effect value	Boot SE	95% Boot CI	Relative effect proportion
Body Image → Eating Disorders (Risk Pathway)				
Total effect	0.656	0.029	[0.599, 0.714]	100%
Direct effect (H1d)	0.293	0.029	[0.236, 0.348]	44.66%
Total indirect effect	0.363	0.030	[0.305, 0.423]	55.34%
Indirect 1: Body image → Thin-ideal internalization → Eating disorders (H1a)	0.081	0.013	[0.055, 0.106]	12.35%
Indirect 2: Body image → Depression → Eating disorders (H1b)	0.073	0.009	[0.055, 0.092]	11.13%
Indirect 3: Body image → Perceived body fat → Eating disorders (H1c)	0.209	0.020	[0.170, 0.248]	31.86%
Body Image → Physical Activity (Adaptive Pathway)				
Total effect	0.336	0.024	[0.288, 0.383]	100%
Direct effect (H2b)	0.216	0.025	[0.167, 0.264]	64.29%
Indirect effect: Body image → Muscular-ideal internalization → Physical activity (H2a)	0.120	0.016	[0.090, 0.152]	35.71%

**Figure 1:**
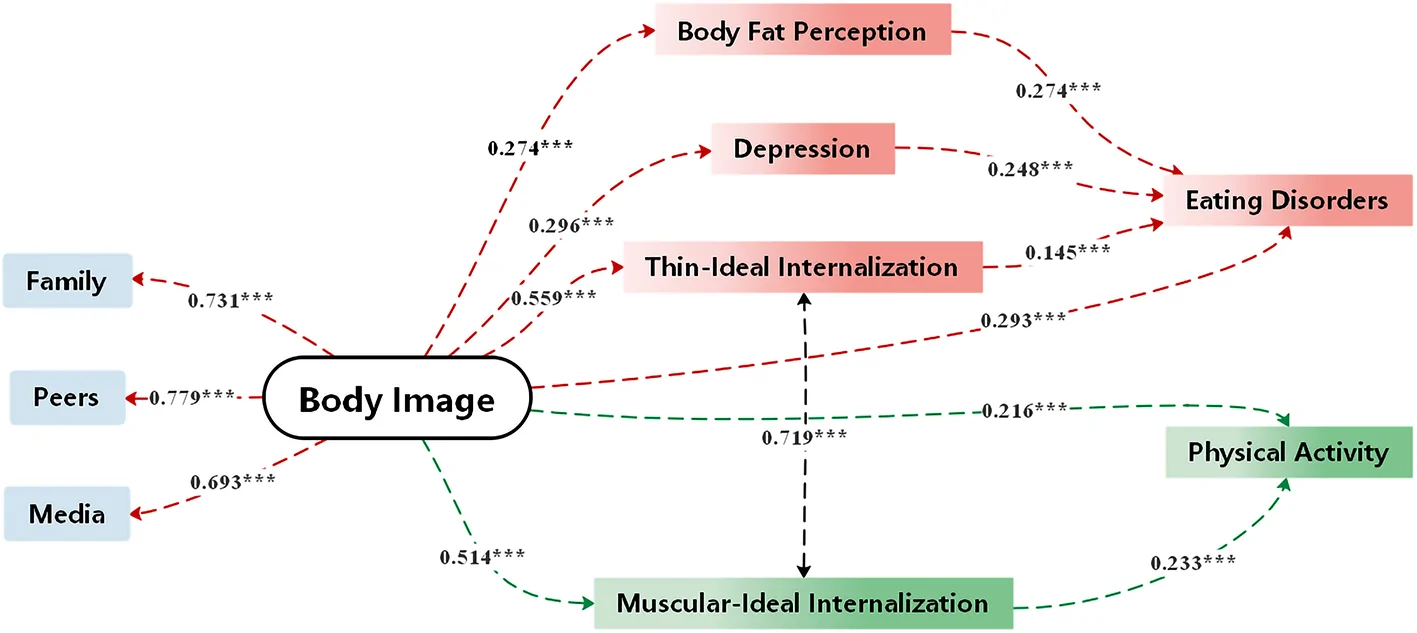
Path coefficient diagram of the dual-pathway differentiation model (conceptually adapted from Tylka, 2011).

### 5.5 Mediating effects in the risk pathway

In the ‘body image → disordered eating' pathway, three mediating pathways through thin-ideal internalization [effect = 0.081, 95% CI (0.055, 0.106), supporting H1a), perceived body fat [effect = 0.209, 95% CI (0.170, 0.248), supporting H1c), and depression [effect = 0.073, 95% CI (0.055, 0.092), supporting H1b) were significant. The combined indirect effect of these mediators accounted for 55.34% of the total effect, indicating partial mediation.

### 5.6 Mediating effects in the adaptive pathway

In the ‘body image → physical activity' pathway, the mediating effect through muscular-ideal internalization was statistically significant [effect = 0.120, 95% CI (0.090, 0.152)], supporting Hypothesis H2a.

### 5.7 Summary of research hypothesis testing

Based on the above analysis, the results of hypothesis testing are summarized as follows.

*Hypothesis 1 (Risk Pathway):* Fully supported: body image showed significant indirect associations with disordered eating via thin-ideal internalization (H1a), depression (H1b), and perceived body fat (H1c), as well as a significant direct association with disordered eating (H1d).

*Hypothesis 2 (Adaptive Pathway):* Fully supported: body image showed a significant indirect association with physical activity via muscular-ideal internalization (H2a), as well as a significant direct association with physical activity (H2b).

*Hypothesis 3 (Exploratory Hypothesis):* Fully supported: Thin-ideal internalization and muscular-ideal internalization demonstrated a strong positive correlation (*r* = 0.719, *p* < 0.01).

## 6 Discussion

This study constructed and tested a dual-pathway mediation model to explore differentiated behavioral outcomes associated with body image among female college students, systematically examining the mediating roles of thin-ideal internalization, muscular-ideal internalization, perceived body fat, and depression between body image and two core outcome variables, disordered eating and physical activity. The results validated the hypothesized pathways and provided new insights into the differentiation mechanism through which body image influences related behaviors, thereby offering significant theoretical and practical implications.

### 6.1 Direct effects of body image on outcome variables

This study demonstrated that body image—as operationalized by family, peer, and media pressures—had significant direct associations with both disordered eating (H1d) and physical activity (H2b). This finding aligns with the core tenet of the sociocultural model that BID is a key antecedent of disordered eating (Shroff and Thompson, 2006) and extends existing theory by revealing a dual-pathway mediation model of body image. Consistent with previous conclusions (Puspita and Estiningtyas, 2024), BID is associated with increased eating disorder risk by reinforcing individuals' cognitive distortions regarding weight and body shape, such as excessive veneration of slim body types and excessive attention to body size. Prior evidence suggests that disordered eating is primarily observed in young women (Sharan and Sundar, 2015), and the degree of BID in adolescent females is directly related to the frequency of eating disorder symptoms (Parmar et al., 2021). Meanwhile, the direct association between body image and physical activity is consistent with findings suggesting that body image can serve as a motivational trigger for exercise behavior (Yuan et al., 2025; Zhou et al., 2024). Additionally, the direct path from body image to disordered eating accounted for 44.66% of the total statistical effect, indicating that although mediating factors play a crucial role, body image itself remains an independent predictor of disordered eating. This suggests that body image relationships with behavioral outcomes may involve interactions with individual and environmental factors, though causal mechanisms remain to be established.

### 6.2 Mediating role of thin-ideal internalization in the risk pathway

This study verified the significant mediating effect of thin-ideal internalization between body image and disordered eating (H1a), supporting the risk pathway mechanism proposed in Hypothesis 1. This finding further corroborates the core mechanism of the sociocultural model, namely that external sociocultural pressures from family, peers, and media are internalized as individuals' slim standards, subsequently triggering eating disorder behaviors (van den Berg et al., 2002). Recent research by Besong (2024) also found that conversations about dieting and BID within families, peer interactions, and media exposure are associated with unhealthy eating behaviors among adolescent females. Jiotsa et al. (2021) further reported that social media usage frequency is significantly associated with adolescents' ‘drive for thinness'. Specifically, female college students with BID are more likely to internalize socially constructed thin ideals (Jiotsa et al., 2021; Chen et al., 2024), leading to cognitive dissonance between actual and ideal body types. To alleviate this dissonance, individuals may adopt extreme weight control strategies such as restrictive eating and binge eating, which are typical manifestations of eating disorders (Ricciardelli and McCabe, 2004). The mediating effect of thin-ideal internalization accounted for 12.35% of the total effect, highlighting its specific role in the risk pathway. This finding is consistent with research by de Carvalho et al. (2017) on Brazilian women, which identified thin-ideal internalization as a mediating variable between body image and eating disorder behaviors.

### 6.3 Mediating roles of perceived body fat and depression in the risk pathway

H1b and H1c were supported: both depression and perceived body fat are significant mediating variables between body image and disordered eating. Among these mediators, perceived body fat exhibited the strongest mediating effect, accounting for 31.86% of the total effect and demonstrating its central position in the risk pathway. Research results showed that BID is strongly associated with individuals' perception of being overweight, and this cognitive pattern also correlates with eating disorder behaviors. This pattern aligns with the information processing model (Beck, 1987), which posits that individuals with BID develop selective attention and over-interpretation of body-related information, thereby forming cognitive biases regarding their perceived degree of obesity (Ralph-Nearman, 2018). Such cognitive biases trigger strong weight-related anxiety, prompting individuals to adopt unhealthy weight control behaviors such as restrictive dieting (Zarychta et al., 2020). Depression represents another important mediating variable linking body image and eating disorders, reflecting the operation of emotional mechanisms. Depressive symptoms may induce emotional eating, defined as alleviating negative emotions through eating, or external eating, characterized by impulsive eating triggered by food cues, and the severity of depression shows a dose–response relationship with these unhealthy eating behaviors (Zhao et al., 2025). Body image dissatisfaction is closely related to difficulties in emotion regulation (Abdoli et al., 2025), and BID positively predicts depressive symptom severity, which in turn further exacerbates eating disorder tendencies (Zhao et al., 2025). This finding highlights the potential bridging role of emotional factors in the associations among body image and disordered eating, though temporal precedence remains unestablished.

### 6.4 Mediating role of muscular-ideal internalization in the adaptive pathway

This study identified a significant mediating role of muscular-ideal internalization between body image and physical activity (H2a established). This finding provides a mechanistic explanation for understanding the specific pathways through which body image influences behavior. Female college students with BID may simultaneously endorse thin and muscular body ideals (Bozsik et al., 2018) and may view the pursuit of muscular definition as a means of improving body function and appearance (Soulliard et al., 2019), thereby promoting physical activity participation and extending the virtuous cycle model of ‘BID reduction → enhanced physical activity' (Gao et al., 2024). The mediating role of muscular-ideal internalization underscores the importance of distinguishing different body ideal types when interpreting behavioral outcomes. Unlike thin-ideal internalization, which is primarily associated with eating disorder risk, muscular-ideal internalization demonstrates adaptive potential related to physical activity engagement. The present findings are consistent with those reported by Ruiz-Bravo et al. (2025) and Du et al. (2023), who similarly documented associations between body image-related processes and physical activity, collectively suggesting that muscular-ideal internalization may play a role in the relationship between body image and exercise behavior. The strong correlation between thin-ideal and muscular-ideal internalization (r = 0.719) warrants further consideration. Rather than representing two entirely independent constructs, this high correlation may reflect a ‘lean and fit' or ‘toned' ideal that has become increasingly prevalent among women, particularly through social media content that simultaneously promotes thinness and muscular definition (Bozsik et al., 2018). This suggests that many women may internalize a blended ideal rather than exclusively pursuing either thinness or muscularity. However, excessive pursuit of the muscular-ideal may also pose potential risks. For example, some individuals may exhibit unhealthy behaviors such as compulsive exercise or misuse of protein supplements and other sports nutrition products due to excessive pursuit of muscular definition and may even develop muscle dysmorphia. Therefore, while encouraging physical activity, attention should also be paid to the potential negative consequences of muscular-ideal internalization, which warrant further exploration in future research.

### 6.5 Theoretical significance of the integrated dual-pathway model

The core theoretical contribution of this study lies in the construction and validation of a dual-pathway differentiation model that explains how body image among female college students diverges into two distinct behavioral trajectories. The coexistence of the risk pathway (body image → thin-ideal internalization/depression/perceived body fat → disordered eating) and the adaptive pathway (body image → muscular-ideal internalization → physical activity participation) helps explain why individuals with similar body image experiences display markedly different behavioral responses. Notably, this study identified a strong positive correlation between thin-ideal internalization and muscular-ideal internalization (H3 established), suggesting that body ideals among female college students may exhibit diversified characteristics, with some individuals simultaneously pursuing both slimness and muscularity. This finding further underscores the complexity of the dual-pathway model, indicating that the same individual may be concurrently influenced by multiple body ideals and thus display risk-oriented or adaptation-oriented behaviors across different contexts.

Furthermore, the model proposed in this study emphasizes the critical role of different body ideal internalization types as the core mechanism underlying behavioral differentiation: thin-ideal internalization is associated with depression, and perceived body fat, which in turn correlate with disordered eating, whereas muscular-ideal internalization is primarily associated with physical activity participation. This dual-pathway model theoretically extends the sociocultural model and body image theory by demonstrating that body image is not a single factor but can lead to risk-oriented or adaptation-oriented behavioral outcomes through different internalization pathways.

Validation of this model provides a new perspective for understanding the complex mechanisms underlying body image effects and offers a testable theoretical framework for subsequent research.

## 7 Practical implications

The dual-pathway differentiation model identified in this study offers several practical implications for intervention, screening, and health promotion. At the intervention level, muscular-ideal internalization may be a relevant target for promoting physical activity among female college students through body appreciation interventions. At the screening level, thin-ideal internalization could serve as a useful indicator for identifying individuals at risk of disordered eating in university mental health settings. At the public health level, campus-based programs might adopt a balanced approach that emphasizes holistic wellbeing, body acceptance, and sustainable health behaviors across diverse body types.

## 8 Research limitations

This study has some limitations that should be addressed in future research.

First, the cross-sectional design limits causal inference. Future research should employ longitudinal designs or experimental approaches to more rigorously verify causal relationships.

Second, self-report bias may affect the accuracy of the results. All variables were measured using self-report questionnaires, which may be subject to social desirability effects or recall bias. Future research could integrate multiple measurement methods, such as behavioral observation, physiological indicators (such as body fat percentage measurement), or peer evaluations, to enhance data objectivity.

Third, sample representativeness requires improvement. This study used convenience sampling, with participants primarily drawn from female college students at six universities, which may not fully represent the national population of female college students. Future research should expand the sampling scope to include women from different regions, different types of universities, and different age groups to enhance the external validity of the findings. Additionally, this study focused exclusively on women; future research should include male participants to explore potential gender differences.

Fourth, uncontrolled confounding variables may affect the results. Although this study controlled for major variables in the statistical model, other unmeasured confounding factors may still exist, such as personality traits, socioeconomic status, and dietary cultural background. Future research should incorporate additional control variables to enhance the explanatory power of the model.

## 9 Conclusion

Through large-sample empirical research, this study examined a dual-pathway model exploring associations between body image, disordered eating, and physical activity among female college students. The findings revealed differential patterns of association: thin-ideal internalization, perceived body fat, and depression were associated with eating disorder behaviors, whereas muscular-ideal internalization was associated with physical activity. Although the cross-sectional design precludes causal inference, this work contributes to body image theory by proposing a testable framework and identifying distinct correlates of maladaptive vs. potentially adaptive behaviors. Longitudinal research is essential to establish causal mechanisms and assess applicability across diverse populations and cultural contexts.

## Data Availability

The raw data supporting the conclusions of this article will be made available by the authors, without undue reservation.
